# Genomic insights of *Pannonibacter phragmitetus* strain 31801 isolated from a patient with a liver abscess

**DOI:** 10.1002/mbo3.515

**Published:** 2017-08-30

**Authors:** Yajun Zhou, Tao Jiang, Shaohua Hu, Mingxi Wang, Desong Ming, Shicheng Chen

**Affiliations:** ^1^ Yun Leung Laboratory for Molecular Diagnostics School of Biomedical Sciences Huaqiao University Xiamen Fujian China; ^2^ Institute of Nanomedicine Technology and Department of Medical Laboratory Weifang Medical University Weifang Shandong China; ^3^ Department of Clinical Laboratory Quanzhou First Hospital Affiliated to Fujian Medical University Fujian China; ^4^ Department of Microbiology and Molecular Genetics Michigan State University East Lansing MI USA

**Keywords:** antibiotic resistance mechanism, comparative genomic analysis, genomic sequencing, *Pannonibacter phragmitetus*, pathogenesis

## Abstract

*Pannonibacter phragmitetus* is a bioremediation reagent for the detoxification of heavy metals and polycyclic aromatic compounds (PAHs) while it rarely infects healthy populations. However, infection by the opportunistic pathogen *P. phragmitetus* complicates diagnosis and treatments, and poses a serious threat to immunocompromised patients owing to its multidrug resistance. Unfortunately, genome features, antimicrobial resistance, and virulence potentials in *P. phragmitetus* have not been reported before. A predominant colony (31801) was isolated from a liver abscess patient, indicating that it accounted for the infection. To investigate its infection mechanism(s) in depth, we sequenced this bacterial genome and tested its antimicrobial resistance. Average nucleotide identity (ANI) analysis assigned the bacterium to the species *P. phragmitetus* (ANI, >95%). Comparative genomics analyses among *Pannonibacter* spp. representing the different living niches were used to describe the *Pannonibacter* pan‐genomes and to examine virulence factors, prophages, CRISPR arrays, and genomic islands. *Pannonibacter phragmitetus* 31801 consisted of one chromosome and one plasmid, while the plasmid was absent in other *Pannonibacter* isolates. *Pannonibacter phragmitetus* 31801 may have a great infection potential because a lot of genes encoding toxins, flagellum formation, iron uptake, and virulence factor secretion systems in its genome. Moreover, the genome has 24 genomic islands and 2 prophages. A combination of antimicrobial susceptibility tests and the detailed antibiotic resistance gene analysis provide useful information about the drug resistance mechanisms and therefore can be used to guide the treatment strategy for the bacterial infection.

## 1 INTRODUCTION

*Pannonibacter phragmitetus* was first found in human blood cultures in the United Kingdom in 1975 (Holmes, Segers, Coenye, Vancanneyt, & Vandamme, 2006). As a novel genus and species, the nomenclature *P. phragmitetus* was given to an alkalitolerant strain C6/19T (=DSM 14782T = NCAIM B02025T) isolated from decomposing reed rhizomes in a Hungarian soda lake in 2003 (Borsodi et al., 2003). *Pannonibacter phragmitetus* is a Gram‐negative, facultative anaerobic, chemoorganotrophic, and motile rod (Borsodi et al., 2003). Before 2006, it was misclassified as *Achromobacter* groups B and E. With 16S rRNA gene sequencing, DNA–DNA hybridization, G+C content determination, cellular fatty acid evaluation, and biochemical experiments, Holmes et al. (2006) verified that *P. phragmitetus* and *Achromobacter* groups (B and E) belonged to same taxon (Holmes et al., 2006). The same group had shown that “Achromobacter groups B and E” are the single genus and species in biochemical characteristics (Holmes et al., 1993).

*Pannonibacter phragmitetus* survives in extreme environments including hot springs (Bandyopadhyay, Schumann, & Das, 2013; Coman, Druga, Hegedus, Sicora, & Dragos, 2013), alkaline environments (pH 7.0–11.0), zero to high salinity (no NaCl as well as up to 5% [w/v] NaCl) (Borsodi et al., 2003). Currently, the studies on *P. phragmitetus* mainly focused on its bioremediation potentials including reduction of heavy metal chromium and detoxification of polycyclic aromatic compounds (PAHs) under extreme conditions (Borsodi et al., 2005; Xu et al., 2011a; Shi et al., 2012; Wang et al., 2013; Wang, Jin, Zhou, & Zhang, 2016). For example, Shi et al. (2012) isolated a strain that could be used for the bioremediation of chromate‐polluted soil and water (Shi et al., 2012). This strain displayed the complete Cr(VI) reduction capability under anaerobic conditions (Shi et al., 2012). Furthermore, Xu et al. (2012) demonstrated that reduction capacity of hexavalent chromium by *P. phragmitetus* LSSE‐09 was not impaired under alkaline conditions (up to pH 9.0) (Xu et al., 2011b). Chai et al. (2009) showed that *P. phragmitetus* BB could maintain an intact cell surface with a strong chromium reduction capacity under a high concentration of Cr(VI) [500 mg L(−1)] (Chai et al., 2009). Bandyopadhyay et al. (2013) isolated a strain resistant to arsenate in a hot‐spring sediment sample that was also alkalitolerant (Bandyopadhyay et al., 2013). However, the metabolism pathways involving chromium were unclear.

Until now, only four cases of *P. phragmitetus* infections in patients have been reported, including a case of replacement valve endocarditis (McKinley, Laundy, & Masterton, 1990), two cases of septicemia (Holmes, Lewis, & Trevett, 1992), and one case of recurrent septicemia (Jenks & Shaw, 1997). With BD BACTEC 9240 automated blood culture system, we isolated *P. phragmitetus* 31801 from the blood sample of a patient with liver abscess (Wang et al., 2017). The patient made a full clinical recovery after 20 days of antibiotic therapy (aerosol inhalation of 1.5 g cefodizime sodium b.i.d and intravenous injection of 0.5 g metronidazole b.i.d) and percutaneous abscess drainage (Wang et al., 2017). Our previous study demonstrated that *P. phragmitetus* was possibly an opportunistic pathogenic bacterium (Wang et al., 2017). However, the pathogenesis and antibiotic resistance mechanisms in *P. phragmitetus* remain unexplored.

Only two draft genomes in *P. phragmitetus* were available in GenBank (CGMCC9175, accession number: LGSQ01000001.1; DSM 14782, accession number: NZ_KB908215.1). There was not enough information available to fully understand the metabolism pathway of heavy metals, the pathogenesis and antibiotic resistance mechanisms. In this article, we first reported the genome sequences from a clinical isolate *P. phragmitetus* strain 31801. Antibiotic resistance genes and virulence factors were investigated. Furthermore, we also provided a genomic insight into the mechanisms of hexavalent chromium reduction at the genomic level.

## 2 MATERIALS and METHODS

### 2.1 Strain and culture conditions

*Pannonibacter phragmitetus* was isolated on Columbia blood agar plates at 37°C. After overnight incubation, a single colony was purified, subcultured in Luria–Bertani (LB) broth, and stored at −80°C for further study.

### 2.2 DNA extraction and whole genome sequencing

Genomic DNA was extracted using Qiagen DNA mini Kit (Qiagen, Germany). The strain identity was first confirmed by 16S rRNA sequencing (GenBank No. FJ882624.1). Genome sequencing was performed with PacBio single‐molecule real‐time (SMRT) RS II technique (Pacific Biosciences, Menlo Park, CA, USA).

### 2.3 Assembly, annotation, and bioinformatics analysis

Raw sequence data processing and genome assembly were performed by SMART portal V2.3. RS_HGAP_Assembly.3 (Pacific Biosciences). Gene calling was finished by using GeneMarkS and Glimmer 3 (Besemer & Borodovsky, 2005; Delcher, Bratke, Powers, & Salzberg, 2007). Initial prediction and annotation of open reading frames (ORF) prediction were carried out with Glimmer 3 using the Rapid Annotation in Subsystem Technology server (RAST) (Delcher, Harmon, Kasif, White, & Salzberg, 1999; Aziz et al., 2008). The quality was further evaluated by GeneMarkS ORF prediction (Besemer & Borodovsky, 2005). DNA sequence visualization and annotation were conducted by Artemis 16 (Rutherford et al., 2000; Carver, Harris, Berriman, Parkhill, & McQuillan, 2012) when necessary. The functional categorization and classification for predicted ORFs were performed by IMG/IMG ER, RAST server‐based SEED viewer, Clusters of Orthologous Groups, and WebMGA programs (Markowitz et al., 2009; Wu, Zhu, Fu, Niu, & Li, 2011; Markowitz et al., 2014; Overbeek et al., 2014; Galperin, Makarova, Wolf, & Koonin, 2015). A circular genome map was generated using GCView based on all predicted CDS information, tRNAs/rRNAs, GC content, and gene cluster information (Grant & Stothard, 2008). The multidrug resistance genes were predicted using both CARD database (McArthur et al., 2013) and RAST (Delcher et al., 1999; Aziz et al., 2008). Prophage and Clustered Regularly Interspaced Short Palindromic Repeats (CRISPR) were predicted by using PHAST (Zhou, Liang, Lynch, Dennis, & Wishart, 2011) and CRISPRfinder (Grissa, Vergnaud, & Pourcel, 2008). Detection and identification of virulent factors were carried out using VFDB database (Chen, Xiong, Sun, Yang, & Jin, 2012). The pan‐genome analysis was conducted by EDGAR 2.0 (Blom et al., 2016). Secondary metabolic analysis was conducted using the server antiSMASH (Weber et al., 2015) version 3.0.5.

### 2.4 The antimicrobial susceptibility test

The antimicrobial susceptibility test (AST) was conducted with the Kirby–Bauer disk diffusion test (K‐B) method (Oxoid, England) (Jorgensen & Ferraro, 2009) and was verified with BD's Phoenix™ 100 Automated Microbiology System with the NMIC/ID‐109 identification/antibiotic susceptibility cards (Becton, Dickinson and Company), according to the NCCLS (Standards, 2006) and CLSI Performance Standards (Institute, 2013). The bacteria was cultured with AST MH broth at 35°C. The positive control was *Pseudomonas aeruginosa* ATCC27853. The threshold used to determine a strain resistant or sensitive was established according to the CLSI Performance Standards (Standards, 2013). AST tests were replicated three times.

### 2.5 Deposition of genome sequences

The genome and plasmid sequences were deposited in the GenBank database (accession no. CP013068 and CP013069). The BioProject designation for this project is PRJNA298840. BioSample accession number is SAMN04158710.

## 3 RESULTS and DISCUSSION

### 3.1 Genomic properties

**Figure 1:**
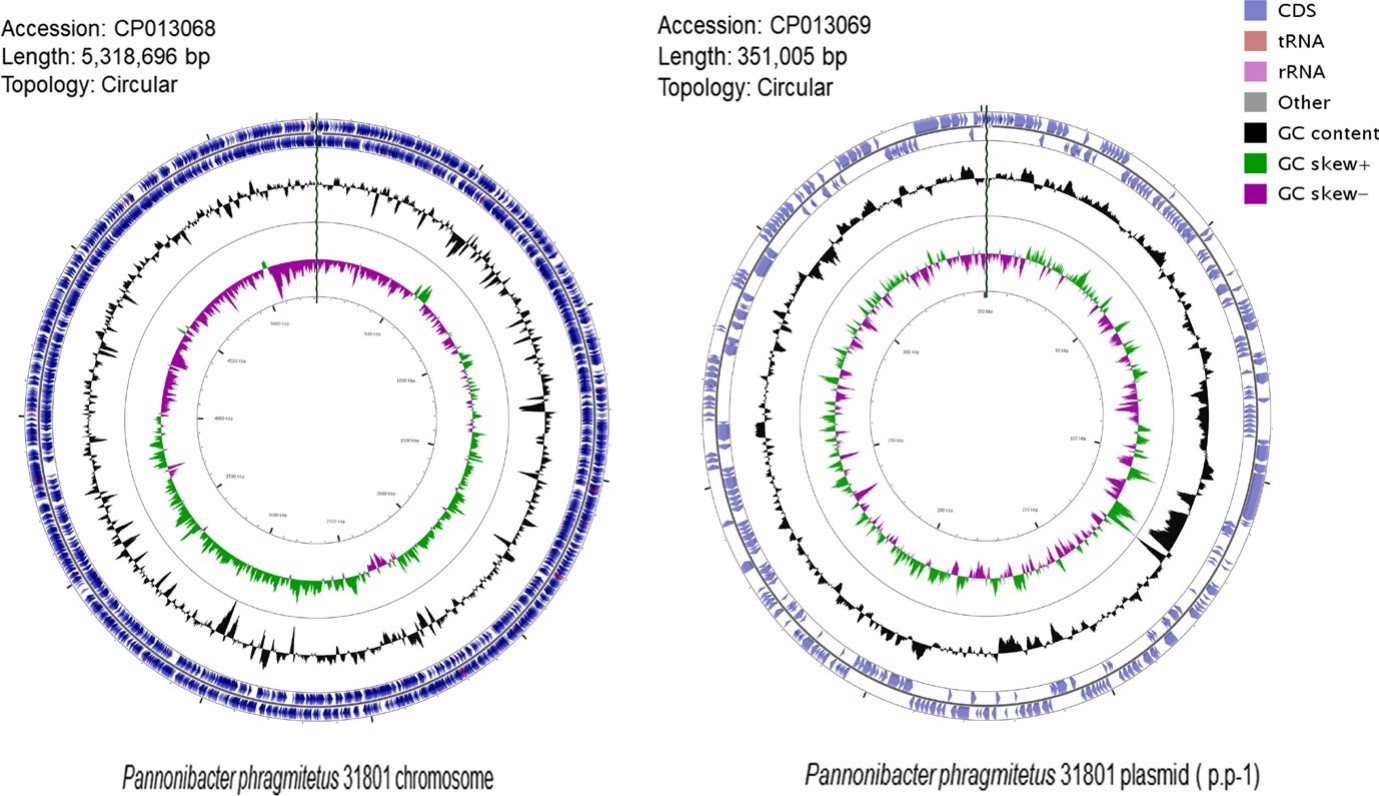
Representation of the completed chromosome and plasmid of *Pannonibacter phragmitetus* 31801. Concentric rings, from outer to inner rings, represent the following features: in the clockwise or counterclockwise direction, the coding sequences (CDS) in light blue, rRNAs in pink, tRNAs brown; GC content (percentage) as a peak to valley profile in black; GC‐skew graph in purple and green

From the sequencing libraries, 1,245,556,320 bp reads were obtained. It was estimated to be at least 163‐fold coverage of the genome. After genome assembly, a complete circular chromosome and a circular plasmid were identified with a size of 5,318,696 and 351,005 bp, respectively (Figure 1). The average GC contents are 63.3% and 63.9% for the chromosome and plasmid, respectively, consistent with those in other *Pannonibacter* genomes (Table 1). The genome size of *P. phragmitetus* strain 31801 is slightly smaller than that in *P. phragmitetus* CGMCC9175, but is much larger than those in *P. phragmitetus* DSM 14782 and *Pannonibacter indicus* 2340 (Table 1). The chromosome contains at least 4,997 protein‐coding genes (Table 1). It has the most rRNA and tRNA gene numbers (9 rRNAs and 54 tRNAs) among the selected genomes (Table 1). It is interesting that all *Pannonibacter* genomes have CRISPRs with the copies ranging from 3 to 6 (Table 1). Furthermore, *P. phragmitetus* strain 31801 possesses a plasmid (p.p‐1) which contains at least 308 genes (see below).

**Table 1:** Genomic features of selected *Pannonibacter* spp. (chromosome)

Strains	*P. phragmitetus*			*P. indicus*
	31801	CGMCC9175	DSM 14782	2340
Accession	CP013068	LGSQ01000001.1	NZ_KB908215.1	NZ_LIPT01000001.1
Size (Mb)	5.32	5.58	4.78	4.17
GC%	63.3	63.6	63.1	63.5
Protein	4,997	4,936	4,122	3,703
rRNA	9	3	4	7
tRNA	54	50	49	50
Gene	5,150	5,060	4,232	3,845
Pseudo gene	81	70	65	84
CRISPR	4	6	3	3
Plasmid	1	0	0	0

### 3.2 Gene repertoire of *Pannonibacter* and phylogenetic placement

**Figure 2:**
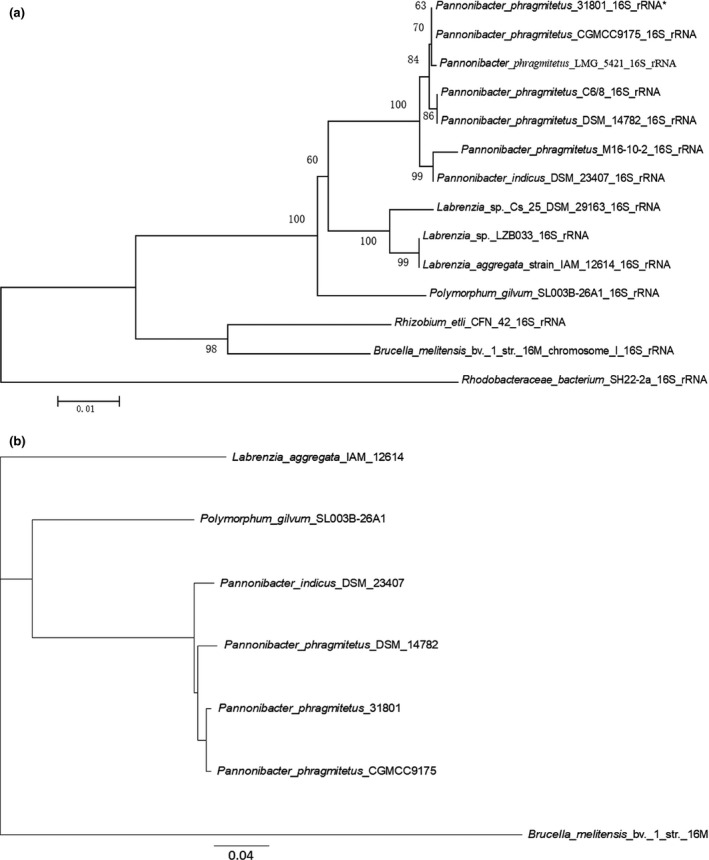
Phylogenetic placement of *Pannonibacter phragmitetus* strain 31801. (a) The genetic placement of *P. phragmitetus* strain 31801 (identity with *) based on 16S rRNA. Alignment of 16S rRNA sequences was conducted using ClustalW (Thompson, Higgins, & Gibson, 1994), and the tree was generated using the neighbor‐joining algorithm with 1,000 bootstraps, using MEGA 6.0. The corresponding GenBank accession numbers were indicated in parentheses. Rhodobacteraceae bacterium SH22‐2a was used as out‐group. (b) Phylogenetic tree inferred from concatenated genes. The tree is calculated from 1,125 core amino acids sequences per genome (15,750 core amino acid sequences). The accession numbers for selected genomes are *P. phragmitetus* 31801 (CP013068), *P. phragmitetus* CGMCC9175 (LGSQ01000001.1), *P. phragmitetus* DSM 14782 (NZ_KB908215.1), *P. indicus* 23407 (NZ_LIPT01000001.1), *Polymorphum gilvum* SL003B‐26A1 (NC_015259), *Labrenzia aggregata* IAM 12614 (NZ_AAUW00000000.1), and *Brucella melitensis* bv. 1 str. 16M (NZ_AHWC01000000)

**Figure 3:**
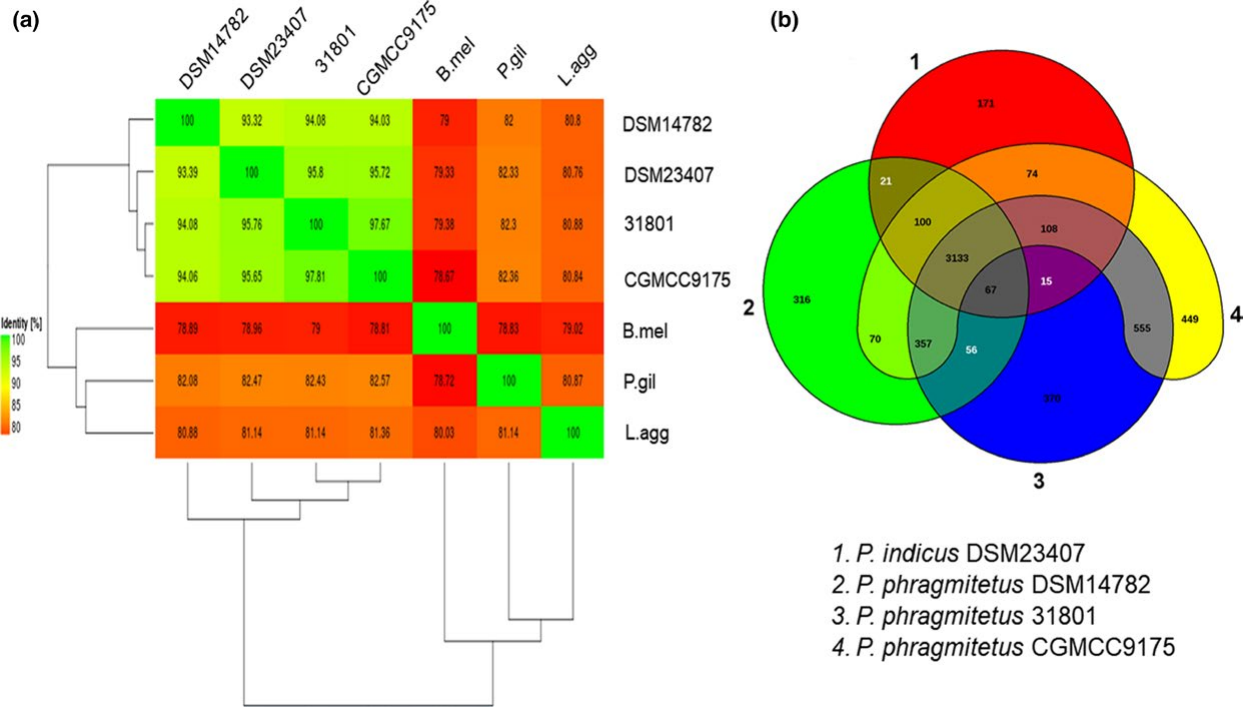
Venn diagram and ANI analysis of representative *Pannonibacter* spp. and their relatives. (a) Heat map of ANI values among *Pannonibacter* spp. and their relatives. 31801 represents *P. phragmitetus* 31801 (CP013068), CGMCC9175 represents *P. phragmitetus* CGMCC9175 (LGSQ01000001.1), DSM 14782 represents *P. phragmitetus* DSM 14782 (NZ_KB908215.1), and DSM 23407 represents *P. indicus* 23407 (NZ_LIPT01000001.1), P.gil represents *Polymorphum gilvum* SL003B‐26A1 (NC_015259), L.agg represents *Labrenzia aggregata* IAM 12614 (NZ_AAUW00000000.1), and B.mel represents *Brucella melitensis* bv. 1 str. 16M (NZ_AHWC01000000). (b) Four representative genomes, *P. phragmitetus* 31801 (CP013068) and *P. phragmitetus* CGMCC9175 (LGSQ01000001.1), *P. phragmitetus* DSM 14782 (NZ_KB908215.1) and *P. indicus* (NZ_LIPT01000001.1) were selected to illustrate the Venn diagram. The Venn diagram was not drawn in proportion; its sole purpose is to illustrate the shared CDSs between the selected strains. The overlapping regions represent CDSs shared with respective strains. The number outside the overlapping regions indicates the number of CDSs in each genome without homologs in other genomes. ANI, average nucleotide identity

Placement of the 16s rDNA sequence from *P. phragmitetus* 31801 into a phylogenetic tree revealed a close relationship with several *P. phragmitetus* isolates from various sources (Figure 2a). However, the 16S rRNA sequence in *P. phragmitetus* 31801 was 100% identical to that in *P. phragmitetus* CGMCC9175, 99% identical to that in *P. phragmitetus* DSM 14782, and 99% identical to that in *P. indicus* DSM 23407, indicating that the 16s rDNA sequence analysis did not provide enough resolution to differentiate *P. phragmitetus* from *P. indicus* (97%, a cut‐off value for the same species; Tindall, Rossello‐Mora, Busse, Ludwig, & Kampfer, 2010). *Pannonibacter phragmitetus* strain 31801 and *P. phragmitetus* CGMCC9175 have an ANI value of 97.67%, agreeing that they belong to the same species according to the microbial taxonomy for species delineation (cut‐off for ANI, 96%; Goris, Konstantinidis, Klappenbach, Coenye, & Vandamme Pand Tiedje, 2007) (Figure 3a). Furthermore, the ANI values between *P. phragmitetus* 31801 genome and *P. phragmitetus* DSM 14782 or *P. indicus* DSM 23407 are below 96%, indicating that they are different species. Overall, *Pannonibacter* genomes are distinctly different from those in *Labrenzia aggregata* and *Polymorphum gilvum* (Figure 2b). An alignment of single‐copy orthologs shared among these selected genomes also shows that *P. phragmitetus* 31801 and *P. phragmitetus* CGMCC9175 clusters more closely, forming a clade. Instead, they branch from *P. phragmitetus* DSM 14782 and *P. indicus* DSM 23407 (Figure 2b).

Comparative genomics analysis was conducted by aligning four available *Pannonibacter* genomes with Progressive Mauve using default parameters (see Figure S1) (Darling, Mau, & Perna, 2010). The genome homology between strain *P. phragmitetus* 31801 and *P. phragmitetus* CGMCC9175 was high, though there were also some rearrangements and sequence elements specific to a particular genome, respectively (see Figure S1). However, the genome synteny between *P. phragmitetus* 31801 and *P. phragmitetus* 23407 was much lower. The gene repertoire of the selected *Pannonibacter* genomes was further analyzed using their ubiquitous genes (core genome) and different homologous gene families (pan‐genome) among the selected *Pannonibacter* and its closest relatives by using EDGAR (Blom et al., 2016). All the four *Pannonibacter* genomes shared a highly conserved genomic architecture as inferred from synteny of protein‐coding orthologs, tRNA genes, rRNA modules, and their origins of replication. An additional 4,153 protein‐coding genes were shared by *P. phragmitetus* 31801 and *P. phragmitetus* CGMCC9175 (Figure 3b). The *P. phragmitetus* 31801 and *P. phragmitetus* CGMCC9175, *P. phragmitetus* DSM 14782, and *P. indicus* genomes had 370, 449, 316, and 171 unique genes, respectively (Figure 3b). Because only four *Pannonibacter* genome sequences are available in GenBank, we added a few bacterial genomes (close relatives) to a “pan vs. core development” curve. The core genome is quite stable at ~3,000 genes for *Pannonibacter* spp. It drops to ~2,400 and ~1,400 when *Po. gilvum*,* L. aggregata*, and *Brucella melitensis* genomes were added (see Figure S2).

### 3.3 Correlation of antibiotic susceptibilities with antibiotic resistance genes

Among the antibiotics tested, *P. phragmitetus* 31801 was susceptible to tazobactam, aztreonam, imipenem, ceftazidime, cefepime, amikacin, gatifloxacin, and levofloxacin (Table 2). Under the same conditions, *P. phragmitetus* 31801 was found to be nonsusceptible to piperacillin (>64 μg/ml), gentamicin (>8 μg/ml), tobramycin (>8 μg/ml), sulfonamides (>2/38 μg/ml), tetracycline (>8 μg/ml), and nitrofurantoin (>16 μg/ml).

**Table 2:** The antimicrobial susceptibility test of *Pannonibacter phragmitetus* strain 31801

Drug class		Antibiotics	MIC (μg/ml)	SIR
β‐lactam antibiotics		Piperacillin/tazobactam	≤2/4	S
	Penicillin class	Piperacillin	>64	R
	Monocyclic β‐lactam	Aztreonam	2	S
	Carbapenems	Imipenem	≤0.5	S
	Cephalosporins	Ceftazidime	4	S
	Cephalosporins	Cefepime	≤1	S
	Cephalosporins	Ceftriaxone	16	I
	Cephalosporins	Cefotaxime	16	I
Aminoglycoside antibiotics		Amikacin	≤8	S
		Gentamicin	>8	R
		Tobramycin	>8	R
Fluoroquinolone antibiotics		Gatifloxacin	≤1	S
		Levofloxacin	≤1	S
Sulfonamides antibiotics		Compound sulfanomides	>2/38	R
Tetracycline antibiotics		Tetracycline	>8	R
Nitrofurans antibiotics		Nitrofurantoin	≤16	R

MIC, minimum inhibitory concentration; SIR, sensitive (S), intermediate (I), resistant (R).

**Figure 4:**
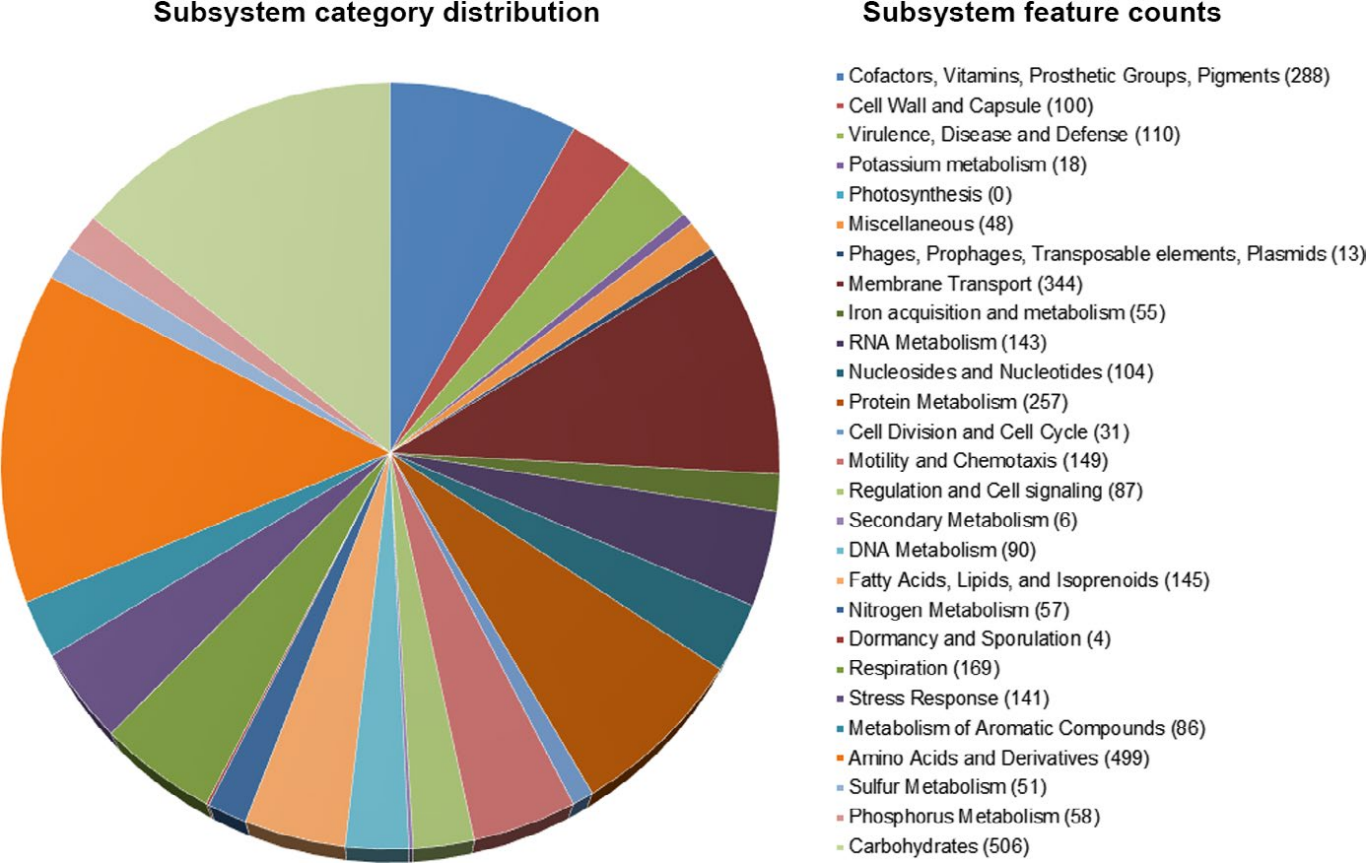
Subsystem distribution in different categories of *Pannonibacter phragmitetus* 31801. Subsystem coverage shows the total genes in the subsystems (49% in subsystems and 51% not in subsystems). Each part of the pie graph indicates different functions and proportions of genes. The numbers in parentheses show the counts of genes with specific functions

The subsystems based on RAST indicates that the annotated genome has up to 110 genes that are potentially involved in virulence, disease, defense, and antimicrobial resistance (Figure 4). They were classified into β‐lactams, fluoroquinolones, fosfomycin, and multidrug resistance efflux pumps (Table S1). Further analysis of the resistance genes in the chromosome genome of *P. phragmitetus* 31801 through the CARD database showed only one β‐lactam resistance gene, NPS β‐lactamase, and more resistance genes involved in multidrug resistance efflux pumps (Table 3). For the latter classification, only *cmeB*,* macA*,* macB*, and *acrB* genes were consistent between the annotated genome (Table S1) and the CARD database (Table 3). However, the fluoroquinolones and fosfomycin resistance genes identified by RAST annotation were not verified through the CARD database.

**Table 3:** The antibiotic resistance genes in *Pannonibacter phragmitetus* 31801 predicted by CARD

Type	Antibiotic resistance ontology	Locus tag	Description/ definition
β‐lactams	NPS β‐lactamase	APZ00_22055	Class A β lactamase found in *Rhodopseudomonas capsulata*.
Multidrug resistance efflux pumps	*acrB*	APZ00_03520, APZ00_24680	Protein subunit of *acrA–acrB–tolC* multidrug efflux complex. *AcrB* functions as a heterotrimer which forms the inner membrane component and is primarily responsible for substrate recognition and energy transduction by acting as a drug/proton antiporter. RND transporter system.
	*acrD*	APZ00_03520, APZ00_24680	*AcrD* is an aminoglycoside efflux pump expressed in *Escherichia coli*. Its expression can be induced by indole, and is regulated by baeRS and cpxAR.
	*acrF*	APZ00_03520, APZ00_24680	*AcrF* is an inner membrane transporter, similar to acrB.
	*adeA*	APZ00_03515	*AdeA* is the membrane fusion protein of the multidrug efflux complex *adeABC*.
	*adeB*	APZ00_03520, APZ00_24680	*AdeB* is the multidrug transporter of the *adeABC* efflux system.
	*adeG*	APZ00_03520, APZ00_24680	*AdeG* is the inner membrane transporter of the *adeFGH* multidrug efflux complex.
	*adeJ*	APZ00_03520, APZ00_24680	*AdeJ* is a RND efflux protein that acts as the inner membrane transporter of the *adeIJK* efflux complex.
	*amrB*	APZ00_03520, APZ00_24680	*AmrB* is the membrane fusion protein of the *amrAB–oprM* multidrug efflux complex.
	*ceoB*	APZ00_03520, APZ00_24680	*CeoB* is a cytoplasmic membrane component of the *ceoAB–opcM* efflux pump
	*cmeB*	APZ00_03520, APZ00_24680	*CmeB* is the inner membrane transporter the *cmeABC* multidrug efflux complex.
	*macA*	APZ00_05170	*MacA* is a membrane fusion protein that forms an antibiotic efflux complex with *macB* and *tolC*.
	*macB*	APZ00_05165	*MacB* is an ATP‐binding cassette (ABC) transporter that exports macrolides with 14‐ or 15‐membered lactones. It forms an antibiotic efflux complex with *macA* and *tolC*.
	*mdsB*	APZ00_03520, APZ00_24680	*MdsB* is the inner membrane transporter of the multidrug and metal efflux complex *mdsABC*.
	*mdtF*	APZ00_03520, APZ00_24680	*MdtF* is the multidrug inner membrane transporter for the *mdtEF–tolC* efflux complex.
	*mexB*	APZ00_01630, APZ00_03520, APZ00_24680	*MexB* is the inner membrane multidrug exporter of the efflux complex *mexAB–oprM*.
	*mexD*	APZ00_03520, APZ00_24680	*MexD* is the multidrug inner membrane transporter of the *mexCD–oprJ* complex.
	*mexF*	APZ00_03520, APZ00_24680	*MexF* is the multidrug inner membrane transporter of the *mexEF–oprN* complex.
	*mexI*	APZ00_01630	*MexI* is the inner membrane transporter of the efflux complex *mexGHI–opmD*.
	*mexY*	APZ00_03520, APZ00_24680	*MexY* is the RND‐type membrane protein of the efflux complex *mexXY–oprM*.
	*mtrD*	APZ00_03520, APZ00_24680	*MtrD* is the inner membrane multidrug transporter of the *mtrCDE* efflux complex.
	*smeB*	APZ00_03520, APZ00_24680	*SmeB* is the inner membrane multidrug exporter of the efflux complex *smeABC* in *Stenotrophomonas maltophilia*.
	*smeE*	APZ00_03520, APZ00_24680	*SmeE* is the RND protein of the efflux complex *smeDEF* in *Stenotrophomonas maltophilia*.
	*tcmA*	APZ00_10885, APZ00_18375	Major facilitator superfamily transporter. Resistance to tetracenomycin C by an active tetracenomycin C efflux system which is probably energized by transmembrane electrochemical gradients.
Mac/lin/phe/str/lin	*cfrA*	APZ00_07870	*Cfr* enzyme adds an additional methyl group at position 8 of A2503 in 23S rRNA, resulting in resistance to florfenicol.

The identity for the resistance genes was above 27.4%. RND, resistance–nodulation–cell division.

AST results showed that *P. phragmitetus* 31801 was susceptible to monocyclic β‐lactam, carbapenems, and cephalosporins, while it was intermediately resistant to cefotaxime (Table 2). The only β‐lactam resistance gene in *P. phragmitetus* 31801 belongs to a class A β‐lactamase, which is known to hydrolyze penicillin (Table 3). It may account for the resistance to piperacillin in our AST test (Table 2). As a matter of fact, we should mention that our patient's liver abscess was cured by abscess draining combined with cefodizime and metronidazole treatment (Wang et al., 2017), the two antibiotics were not included in the AST. It was reported that cefodizime has immunomodulatory properties in stimulating the phagocyte bactericidal function and increasing lymphocyte responses (Labro, 1992). We could not give a clear answer about which strategy(s) played a critical role in curing our patient's infection.

Efflux pumps in Gram‐negative bacteria are exporters of various antimicrobial compounds and biological metabolites (May, 2014). For the genes encoding for the multidrug resistance efflux pumps in *P. phragmitetus* 31801 obtained with CARD analysis, they could confer the resistance to four types of antibiotics: (1) tetracycline: *acrB*,* adeA*,* adeB*,* adeG*,* adeJ*,* mexB*,* mexY*,* smeE*; (2) fluoroquinolone: *acrB*,* acrF*,* adeG*,* adeJ*,* ceoB*,* cmeB*,* mdtF*,* mexB*,* mexD*,* mexF*,* mexI*,* mexY*,* smeB*,* smeE*; (3) aminoglycoside: *acrD*,* amrB*,* ceoB*,* mexY*,* smeB*; (4) macrolide: *adeJ*,* cmeB*,* macA*,* macB*,* mdtF*,* mexB*,* mexD*,* mexY*,* smeE*,* cfrA*. All of them encoded the subunits of the efflux pump.

It was reported that the tripartite efflux system (*acrA‐acrB‐tolC*) led to tetracycline resistance in *Escherichia coli* (Piddock, 2006), while overproduction of *acrA‐acrB‐tolC* contributed to clinical fluoroquinolone resistance (Swick, Morgan‐Linnell, Carlson, & Zechiedrich, 2011). *acrB*, as an inner membrane component of the resistance nodulation cell division (RND) family, was verified to be the major site for substrate recognition and energy transduction in the tripartite efflux system (Pos, 2009). The tripartite efflux system *acrA‐acrB‐tolC* in *P. phragmitetus* 31801 is incomplete because only *acrB* and *tolC* are identified in the genome. We assume that other genes including *adeA*,* adeB*,* adeG*,* adeJ*,* mexB*,* mexY*, or *smeE* might contribute to the tetracycline resistance.

Our strain 31801 was sensitive to amikacin, but resistant to gentamicin and tobramycin (Table 2). The quinolones' resistance generally requires overexpression of *acrA‐acrB‐tolC*. The sensitivity to the fluoroquinolone antibiotics can thus be explained by the fact that *acrA* is not identified by the RAST and CARD database. The *adeABC* system was shown to pump out amikacin, chloramphenicol, cefotaxime, erythromycin, gentamicin, kanamycin, norfloxacin, netilmicin, ofloxacin, pefloxacin, sparfloxacin, tetracycline, tobramycin, and trimethoprim (Magnet, Courvalin, & Lambert, 2001). The strain 31801 contains only gene *adeA*,* adeB*, which gives less clues for explaining the AST in strain 31801. The *adeIJK* pump efflux is involved in the resistance to β‐lactams, chloramphenicol, tetracycline, erythromycin, lincosamides, fluoroquinolones, fusidic acid, novobiocin, rifampicin, trimethoprim, acridine, pyronin, safranin, and sodium dodecyl sulfate (SDS) (Damier‐Piolle, Magnet, Bremont, Lambert, & Courvalin, 2008). There was a synergistic interplay between *adeIJK* and *adeABC* for resistance to chloramphenicol, fluoroquinolones, and tetracyclines (Coyne, Courvalin, & Perichon, 2011). The gene *adeJ* in strain 31801 has 57% identity with that in *E. coli acrB* (Damier‐Piolle et al., 2008). Deletion of the *adeFGH* pump in a mutant strain of *Acinetobacter baumannii* lacking the *adeABC* and *adeIJK* pumps was further shown to confer hypersusceptibility to chloramphenicol, trimethoprim, ciprofloxacin and clindamycin (Coyne, Rosenfeld, Lambert, Courvalin, & Perichon, 2010). Only *adeG* was found in strain 31801 (Table 3). These data could not help to clarify why strain 31801 was sensitive to amikacin while was resistant to gentamicin and tobramycin.

The genome of strain 31801 contains *mexY* and *acrB* (Table 3). It was suggested that, in *Ps. aeruginosa*,* mexY* promoted aminoglycoside resistance (Lau, Hughes, & Poole, 2014). In the same study, the proximal binding pocket within *mexY* was jointed with a periplasm‐linked cleft and was also part of a drug efflux pathway of *acrB*, which conferred to the resistance (Lau et al., 2014). It was verified that overexpression of *mexXY* in *Ps. aeruginosa* promoted resistance to aminoglycoside (Raymond, Dertz, & Kim, 2003) and *mexY* could be induced by chloramphenicol, tetracycline, macrolides, and aminoglycosides (Jeannot, Sobel, Farid, Keith, & Patrick, 2005). The gene *mexY* in strain 31801 genome may have the similar functions in conferring resistance to these antibiotics (Table 3). *SmeB* and *mexB* in strain 31801 showed 52% identity to *mexY* (Li, Zhang, & Poole, 2002). In Gram‐negative bacteria, when *smeABC* multidrug efflux system was overexpressed, the bacteria became more resistant to aminoglycosides, β‐lactams, and fluoroquinolones (Li et al., 2002). In *Burkholderia vietnamiensis*, the *amrAB‐oprM* efflux system contributed to clinical and in vitro resistance to aminoglycosides tobramycin and azithromycin (Westbrock‐Wadman et al., 1999). Besides *mexY*,* smeB*, and *amrB*, two other aminoglycoside resistance genes *acrD* and *ceoB* were identified in *P. phragmitetus* 31801. However, these data could not help understand why strain 31801 was sensitive to amikacin while it was resistant to gentamicin and tobramycin.

The tripartite efflux pump *macAB–tolC* was involved in antibiotic resistance in Gram‐negative bacteria with *macB* as a basic member in the macrolide transporters family (Lu & Zgurskaya, 2012). Similar efflux pump systems, including *macA*,* macB*, and *tolC*, were found in strain 31801 (Table 3). It was reported that the resistance gene *tcm*A of tetracenomycin C in *Streptomyces glaucescens* could be induced by the tetracenomycin C itself (Guilfoile & Hutchinson, 1992). The gene *cfrA* in a plasmid pSCFS1 mediated the resistance to clindamycin, macrolides, lincosamides, and streptogramin B (Kehrenberg, Aarestrup, & Schwarz, 2007). The macrolide was not tested in AST; however, the above macrolide resistance‐related genes identified with CARD analysis might promote the resistance to the macrolide. The above multidrug resistance genes available in strain 31801 may contribute to the related antimicrobial resistance. However, further experiments are warranted.

### 3.4 The genes involved in the reduction of hexavalent chromium

RAST annotation shows that there are list of six genes involving in resistance to chromium compounds, including chromate resistance protein chrI, chrB, chromate transport protein *chrA*, rhodanese‐like protein *chrE*, superoxide dismutase SodM‐like protein *chrF*, and superoxide dismutase *chrC*. Only *chrA* gene is found in *P. phragmitetus* 31801 chromosome and plasmid. However, its gene product was not a reductase in *P. phragmitetus* LSSE‐09 (Xu et al., 2011b). It was reported that the expression of *chrB* and *chrA* genes in a chromium‐sensitive *Ochrobactrum tritici* strain resulted in a high chromium resistance (Branco et al., 2008)

### 3.5 Virulence factors

The protein‐encoding genes were searched against the virulence factor database (VFDB) and PATRIC (Table 4). Up to 16 putative virulence factors were identified with high identity to those well‐known ones (Table 4). For example, pyochelin synthetase, flagellar M‐ring protein, peptide synthase, and cyclolysin secretion ATP‐binding protein were found. This bacterium was predicted to have mobility ability including flagellum that enables the bacterial movement and chemotaxis (149 genes predicted in RAST subsystem). When pathogens move to target cells and access to receptors, they directly utilize flagellin to adhere to and colonize on the surface of epithelial cells (Haiko & Westerlund‐Wikstrom, 2013). In many pathogenic bacteria, flagellin or the flagellar proteins have been demonstrated to function as adhesins (Haiko & Westerlund‐Wikstrom, 2013). Besides the function involving in motility and invasion organelles, the flagellum has been demonstrated to not only promote innate immunity, but also function as a predominant cue to prime the adaptive immune response (Dingle, Mulvey, & Armstrong, 2011).

**Table 4:** Virulence factors in *Pannonibacter phragmitetus* 31801 chromosome

Name	Function	Locus tag
*fleQ*	Transcriptional regulator FleQ	APZ00_13230
*fliF*	Flagellar M‐ring protein FliF	APZ00_17970
*fecE*	ATP‐binding protein FecE	APZ00_03600; APZ00_09395
*cyaB*	Cyclolysin secretion ATP‐binding protein.	APZ00_22975; APZ00_10075
*clpV*	Clp‐type ATPase chaperone protein.	APZ00_15745
*tagT*	Type VI secretion associated protein TagT, ATP‐binding component of ABC transporter.	APZ00_21510
*clpV1*	Type VI secretion system AAA+ family ATPase	APZ00_15605; APZ00_22570
*bcrD*	Type III secretion system LcrD homolog protein BcrD	APZ00_21695; APZ00_22570
*pchI*	ABC transporter ATP‐binding protein	APZ00_20870
*fliI*	Flagellum‐specific ATP synthase FliI	APZ00_19800
*algB*	Two‐component response regulator AlgB	APZ00_12920; APZ00_19550; APZ00_21280
*fliP*	Flagellar biosynthetic protein FliP	APZ00_09865
*pchF*	Pyochelin synthetase PchF	APZ00_05615
*pchH*	ABC transporter ATP‐binding protein	APZ00_02420
*fha1*	Type VI secretion system forkhead‐associated protein Fha1	APZ00_12570
*waaG*	B‐band O‐antigen polymerase	APZ00_07670

*Cyab* encoded cyclolysin secretion protein which participates in secretion of CyaA (Table 4) (Glaser, Sakamoto, Bellalou, Ullmann, & Danchin, 1988). Toxin CyaA was a bifunctional protein with adenylate cyclase and hemolytic activities, critical for pathogen *Bordetella pertussis* to colonize in respiratory tract (Glaser et al., 1988; Gross, Au, Smith, & Storm, 1992). Besides CyaB, two different hemolysins (ALV25768.1 and ALV26033.1) may be involved in erythrocyte degradation.

Cyclic di‐GMP phosphodiesterase was predicted here as a virulence factor, participating in synthesis of an important intracellular signaling molecule c‐di‐GMP (Romling, Galperin, & Gomelsky, 2013) (Table 4). Cyclic di‐GMP as the second message was involved in regulation of a variety of cellular functions including motility, autoregulation, flagellum synthesis, biofilm formation, cell invasion, and virulence (Sondermann, Shikuma, & Yildiz, 2012). For example, the disruption of *cdpA* in *Burkholderia pseudomallei* led to a threefold reduction in invasion of human lung epithelial cells and a sixfold decrease in cytotoxicity on human macrophage cells, indicating that *cdpA* contribute to virulence of pathogenic bacteria (Lee, Gu, Ching, Lam, & Chua, 2010).

Some extracellular virulence factors (proteins) require to be secreted through type VI secretion system (T6SS) (Table 4). At least three genes (*fha1*,* clpV1*, and *tagT*) encode virulence proteins that are part of the T6SS secretion machinery. The T6SS mimics the injection apparatus of contractile tailed bacteriophages (Leiman et al., 2009). The type VI secretion system participates in interbacterial competition and animal pathogenesis (Basler, 2015). In *Vibrio cholerae*, mutants deficient in Hcp and VgrG proteins had decreased virulence and infectivity (Pukatzki, Ma, Revel, Sturtevant, & Mekalanos, 2007). Similarly, *Ps. aeruginosa* secreted and transferred Hcp into target cells and chronic infection through the T6SS (Hood et al., 2010). The predicted virulence gene *bcrD* encodes a protein component in type III secretion system (T3SS) which is essential for its pathogenicity (the ability to infect) (Fauconnier et al., 2001). Defects in the T3SS may render a nonpathogenic bacterium (Coburn, Sekirov, & Finlay, 2007). As a matter of fact, there is a type IV secretion system (T4SS) in this genome which may be also important for secretion of virulence factors (see next).

Three genes (*PvdL*,* pchF*, and *pvdI*) involved in siderophore synthesis participate in acquiring iron from the environment (Table 4). They were verified by using the server antiSMASH (Weber et al., 2015) version 3.0.5 (https://antismash.secondarymetabolites.org/). Further examination of *P. phragmitetus* genome reveals at least 36 genes are related with siderophore synthesis, assembly, and transportation (predicted by RAST subsystem). It seems that *P. phragmitetus* may secrete both enterobactin (four genes) and aerobactin (nine genes) which have the capability to chelate a very low concentration of environmental ferric ion (Fe^3+^) with the extreme high affinity (Miethke & Marahiel, 2007). At least 12 ferric ion ABC uptake receptors were identified, which allowed to efficiently transport the chelated and/or free forms of iron from the environment. Furthermore, heme, hemin uptake, and utilization systems were found (32 genes), indicating that *P. phragmitetus* has a sophisticated iron/heme acquisition system.

### 3.6 Plasmids, prophages, and genomic islands

**Figure 5:**
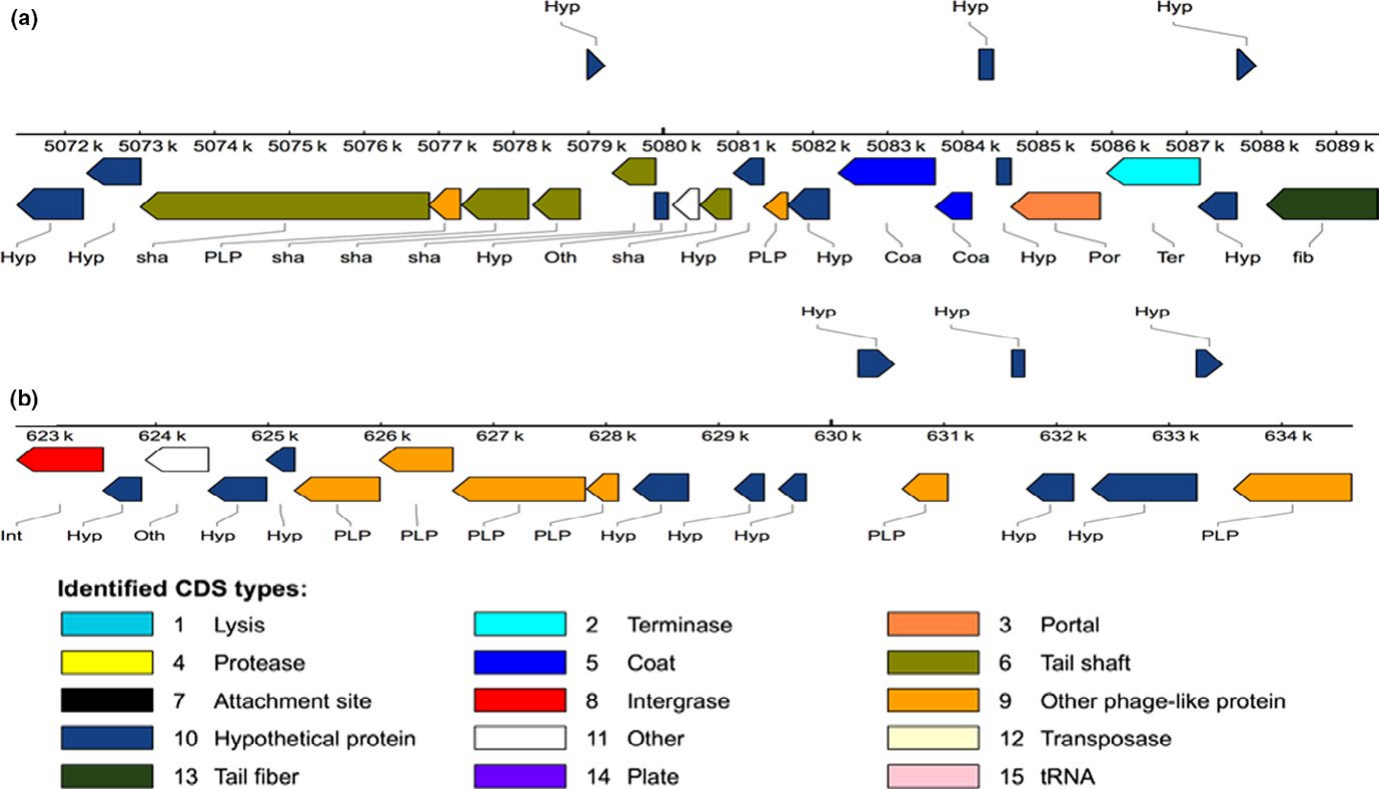
The gene organization of the predicted prophages in *Pannonibacter phragmitetus* 31801 chromosome. (a) The prophage 1 locates between 5,071,359 and 5,089,611 bp (18,253 bp); the GC content is 66.19%. The different colored rectangles indicate the different phage elements. (b) The prophage 2 locates between 622,766 and 633,573 bp (10,807 bp); the GC content is 64. 09%

At least two prophages were identified in *P. phragmitetus* 31801 by using PHAST tool. The prophage 1 is moderately large with a size of 18,253 bps (locates between 5071,359 and 5089,611 bp). The GC content (66.19%) was more than that in average genome (63.3%), indicating that the prophage was horizontally transferred from other microorganisms (Figure 5a). The prophage 1 consists of 21 genes encoding 14 proteins with known functions, 7 hypothetical proteins, and 2 bacterial protein (Figure 5a and Table S2). As predicted by PHAST, prophage 1 was possibly a complete one because it consists of phage tail, head, portal, integrase, lysin, and other component proteins involving in phage structure and assembly. It is interesting that prophage 1 are conserved in three other *Pannonibacter* genomes (>95% identical) and the same prophage also exists in *Po. gilvum* SL003B‐26A1, *Stappia* sp. ES.058, and *Roseibium hamelinense* ATCC BAA‐252 (data not shown), indicating that it is integrated and adopted in the Rhodobacteraceae genomes. Despite that there were no virulence factors encoding genes identified inside the prophage region, we found that some genes encoding penicillin‐binding protein 1A, serine protease, and aminopeptidase immediately up or downstream regions. Not like prophage 1, the second predicted prophage 2 (located between 622,766 and 634,619 bp) seems to be incomplete because many phage structure proteins, lysin, integrase, protease, and transposase genes are absent (Figure 5b, Table S2). It consists of 18 proteins with 9 phage proteins and 9 hypothetical proteins. However, prophage 2 was only found in *P. phragmitetus* 31801 and *P. phragmitetus* CGMCC9175 (data now shown).

**Figure 6:**
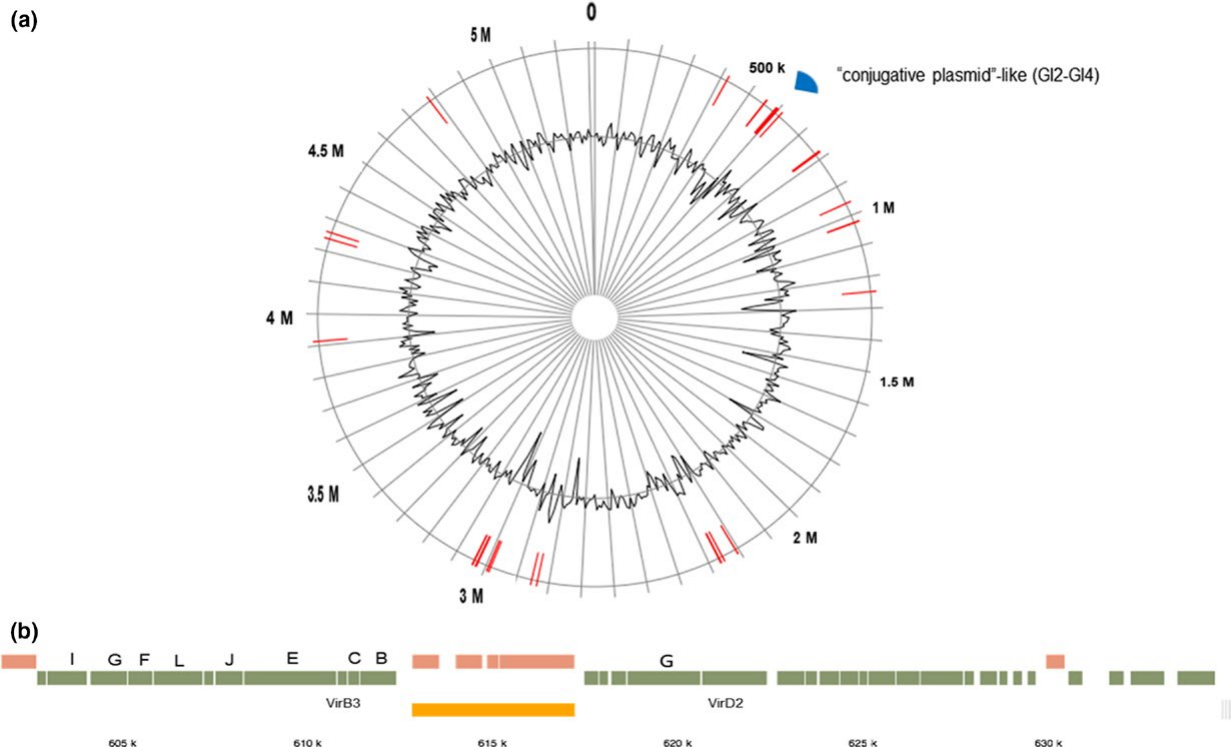
Genomic islands predicted by IslandViewer 3 and the type IV secretion system in *Pannonibacter phragmitetus* 31801 chromosome. (a) Twenty‐four genome islands were predicted with the sizes ranging from 4,202 to 12,259 bp; the “conjugative plasmid”‐like regions (551,959–612,947 bp) span GI and GI4. (b) The putative genetic elements in T4SS shown on the top are Tra I, G, F, L, J, E, C, B, G; virB3 and virD2 are shown below. Their relative positions are shown

Up to 21 genomic islands were found with the sizes ranging from 4,202 to 12,259 bp, sparsely spreading in *P. phragmitetus* 31801 genome (see Figure 6 and Table S3). Further analysis shows that GI2–GI4 regions have a large “conjugative plasmid”‐like genetic element with an estimated size of 60,989 bp (551,959–612,947) (Figure 6). This conjugative plasmid consists of integrases, transposases, transcriptional regulators, and conjugal transfer proteins (type IV secretion systems). Type IV secretion systems (601,481–612,947) exist in *P. phragmitetus* CGMCC9175, while they are absent in *P. phragmitetus* DSM 14782 and *P. indicus*. The function of The type IV secretion systems (TraI, G, F, L, J, E, virB3, C, B, G, and virD2) is well established in Gram‐negative bacteria. Pathogenic Gram‐negative bacteria utilized type IV secretion systems to translocate effector proteins or oncogenic DNA into eukaryotic host cells. Genetic materials can be exchanged through type IV secretion systems, which mediate horizontal gene transfer (Wallden, Rivera‐Calzada, & Waksman, 2010). Thus, type IV secretion systems significantly facilitate the adaptation to dramatic environmental changes which confer the spread of antibiotic resistance among microorganisms (Zechner, Lang, & Schildbach, 2012). Other highlights from these regions are the presence of several genes that are involved in detoxification of heavy metals including arsenic transporters, manganese transporter, zinc transporter ZitB, lead/cadmium/zinc, and mercury transporting ATPase and copper oxidase, which agrees that *P. phragmitetus* has good heavy metal resistance (Xu et al., 2011a; Shi et al., 2012).

The plasmid p.p‐1 has many important functional genes with a total size of 351,005 bp (Figure 1). For example, there are four genes encoding selenate and selenite transporters SomE, F, G, and K, which have been demonstrated to detoxify selenate compounds (based on RAST subsystem analysis). The toxin–antitoxin systems (*parD*,* doc*1, and *doc*2) exists in this p.p‐1, possible participating in stabilization of plasmid and regulation of toxins. Nine siderophore synthesis genes are involved in the formation of enterobactin and aerobactin, indicating that this plasmid is critical for iron uptake. Furthermore, the subsystem analysis (RAST) also showed there are at least 18 genes encoding membrane transporters contributing to the transportation of various nutrient molecules. Therefore, plasmid p.p‐1 in *P. phragmitetus* is one of the largest ones with many important functions among the sequenced Rhodobacteraceae.

## 4 CONCLUSIONS

*Pannonibacter phragmitetus* 31801 is a multidrug‐resistant opportunistic pathogen. From the genomic level, we explained its infection potential by showing that it contained many genes encoding for virulence factors, and its characteristic of multidrug resistance by finding that it contained a list of genes conferring resistance to several classifications of antibiotics. This complete sequenced genome could be the new reference for *P. phragmitetus*. It contributes to further elucidate antibiotic resistance and infectivity mechanisms. It may help understand the evolution traits from bioremediation reagents to virulence strains.

## ETHICS STATEMENT

This article does not contain any studies with human participants or animals performed by any of the authors.

## CONFLICT OF INTEREST

None declared.
