# Supplementary material for: Genomic insights of Pannonibacter phragmitetus strain 31801 isolated from a patient with a liver abscess
Source: Microbiologyopen. 2017 Aug 30;6(6):e00515. doi: 10.1002/mbo3.515 (PMC5727363; doi:10.1002/mbo3.515)
Supplement: Supplementary file 1 [file MBO3-6-na-s001.pdf]

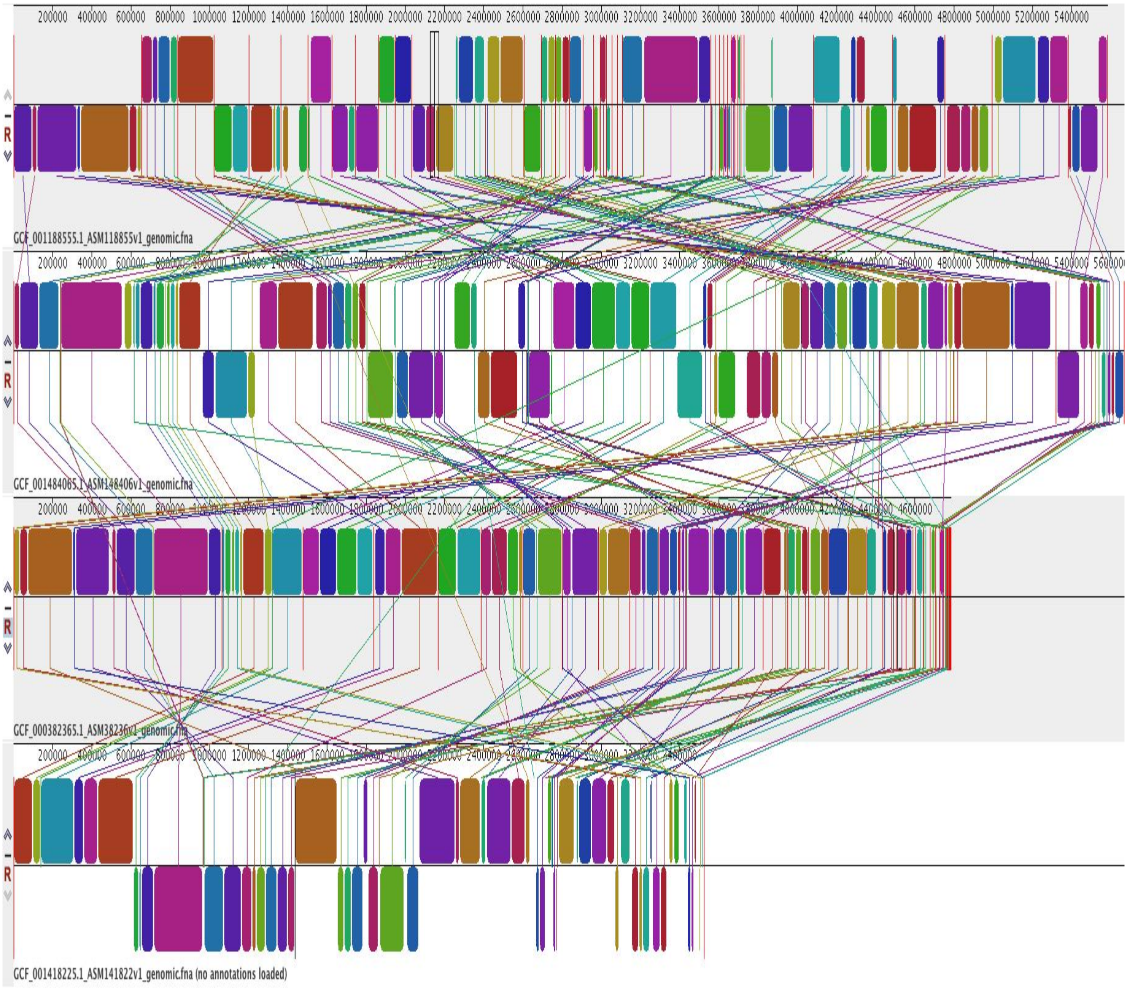

Figure S1. Genomic alignment of the four compared *Pannonibacter* spp.
Alignment statistics were generated and rendered by MAUVE progressive alignment software. S *P. phragmitetus* 31801 (CP013068) and *P. phragmitetus* CGMCC9175 (LGSQ01000001.1), *P. phragmitetus* DSM 14782 (NZ_KB908215.1) and *P. indicus* (NZ_LIPT01000001.1). Color schemes represent blocks of contiguous genes interrupted by colorless patches where the genomes differ from each other significantly and identified as GIs are located.

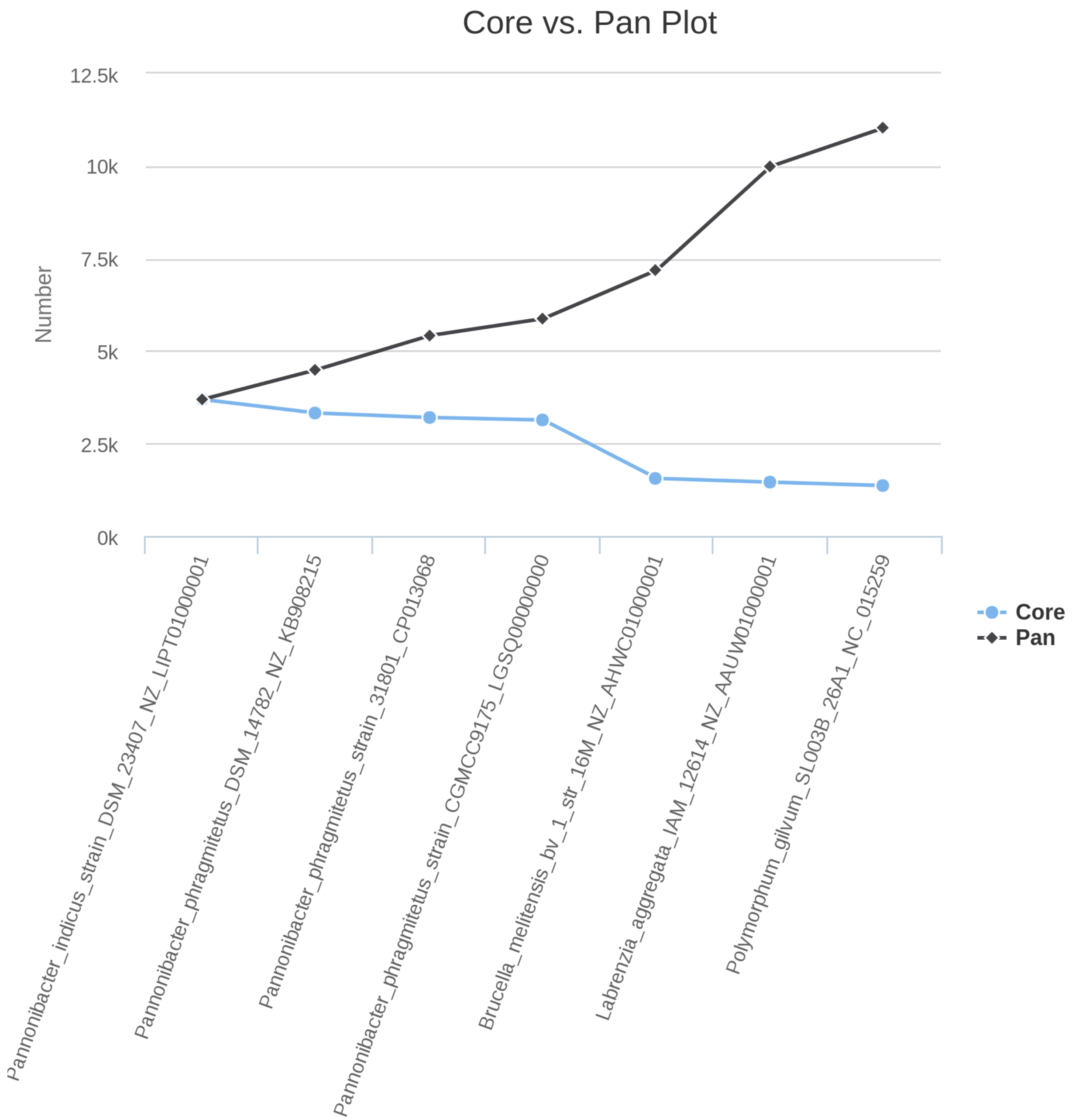

Figure S2. The core vs pan genome development curve. The accession numbers for selected genomes are: *P. phragmitetus* 31801 (CP013068), *P. phragmitetus* CGMCC9175 (LGSQ01000001.1), *P. phragmitetus* DSM 14782 (NZ_KB908215.1), *P. indicus* 23407 (NZ_LIPT01000001.1), *Polymorphum gilvum* SL003B-26A1 (NC_015259), *Labrenzia aggregata* IAM 12614 (NZ_AAUW00000000.1), *Brucella melitensis* bv. 1 str. 16M (NZ_AHWC01000000). B) Four representative genomes, *P. phragmitetus* 31801 (CP013068) and *P. phragmitetus* CGMCC9175 (LGSQ01000001.1), *P. phragmitetus* DSM 14782 (NZ_KB908215.1) and *P. indicus* (NZ_LIPT01000001.1)
