# Supplementary material for: Genomic insights of Pannonibacter phragmitetus strain 31801 isolated from a patient with a liver abscess
Source: Microbiologyopen. 2017 Aug 30;6(6):e00515. doi: 10.1002/mbo3.515 (PMC5727363; doi:10.1002/mbo3.515)
Supplement: Supplementary file 2 [file MBO3-6-na-s002.docx]

**Table S1 Antibiotic and toxic compound resistance predicted by RAST SEED system**

| Drug class | Gene name, description | Locus tag |
| --- | --- | --- |
| β-lactams | BL, β-lactamase (EC 3.5.2.6); | APZ00_22055 |
|  | *BLc, β-lactamase class C and other penicillin binding proteins; | APZ00_20565 |
|  | BLI, Metal-dependent hydrolases of the β-lactamase superfamily I. | APZ00_18330 |
| Fluoroquinolones | ParC, Topoisomerase IV subunit A (EC 5.99.1.-); | APZ00_22160 |
|  | ParE, Topoisomerase IV subunit B (EC 5.99.1.-); | APZ00_19045 |
|  | GyrA, DNA gyrase subunit A (EC 5.99.1.3); | APZ00_18630 |
|  | GyrB, DNA gyrase subunit B (EC 5.99.1.3). | APZ00_06210 |
| Fosfomycin | FosX, Fosfomycin resistance protein FosX. | APZ00_08120 |
| Efflux Pumps | CmeA, RND efflux system, membrane fusion protein CmeA; | APZ00_23425; APZ00_03515 |
|  | CmeB, RND efflux system, inner membrane transporter CmeB; | APZ00_03520 |
|  | *TolC, Type I secretion outer membrane protein, TolC precursor; | APZ00_05175; APZ00_21430 |
|  | *MATE_family_MDR_Pump, Multidrug and toxin extrusion (MATE) family efflux pump YdhE/NorM, homolog; | APZ00_09365 |
|  | MacA, Macrolide-specific efflux protein MacA; | APZ00_05165 |
|  | MacB, Macrolide export ATP-binding/ permease protein MacB (EC 3.6.3.-); | APZ00_05170 |
|  | OML, RND efflux system, outer membrane lipoprotein, NodT family; | APZ00_10065 |
|  | AcrB, Acriflavin resistance protein; | APZ00_12875; APZ00_15245; APZ00_01630; APZ00_18640 |
